# Morpho-Molecular Assessment Indicates New Prognostic Aspects and Personalized Therapeutic Options in Sinonasal Melanoma

**DOI:** 10.3390/cancers11091329

**Published:** 2019-09-07

**Authors:** Sandra N. Freiberger, Grégoire B. Morand, Patrick Turko, Ulrich Wager, Reinhard Dummer, Martin Hüllner, David Holzmann, Niels J. Rupp, Mitchell P. Levesque

**Affiliations:** 1 Department of Pathology and Molecular Pathology, University Hospital Zurich, 8091 Zurich, Switzerland (U.W.) (N.J.R.); 2 University of Zurich, 8006 Zurich, Switzerland (G.B.M.) (P.T.) (R.D.) (M.H.) (D.H.) (M.P.L.); 3 Department of Otorhinolaryngology, Head and Neck Surgery, University Hospital Zurich, 8091 Zurich, Switzerland; 4 Department of Dermatology, University Hospital Zurich, 8091 Zurich, Switzerland; 5 Department of Nuclear Medicine, University Hospital Zurich, 8091 Zurich, Switzerland

**Keywords:** sinonasal melanoma, next generation sequencing, mutations, morpho-molecular assessment, personalized therapy

## Abstract

Sinonasal melanoma is a rare subtype of melanoma and little is known about its molecular fingerprint. Systemic treatment options are limited, as targetable *BRAF* mutations are rare compared to cutaneous melanoma. Currently, metastatic sinonasal melanoma is being treated according to the guidelines of cutaneous melanoma. In this study, we investigated the molecular profile of 19 primary sinonasal melanomas, using a novel customized melanoma-specific next generation sequencing (NGS) panel (MelArray) of 190 genes. Results were correlated to histological and clinical features to further characterize this rare, aggressive type of melanoma and screen for prognostic markers and possible treatment options. Molecular profiles encompassed predominantly mutations in *NRAS* (25%), whereas *KIT* or *BRAF* p.V600 mutations were not detected. Tumor mutational burden was overall low. High level of copy number variations (CNVs) were associated with alterations in DNA-repair genes and shorter distant metastasis-free survival (*p* = 0.005). Monomorphic (vs. pleomorphic) morphology was found to be significantly associated with worse disease-specific survival (*p* < 0.001), however no correlation between morphology and molecular aberrations was found. A variety of alterations in different pathways were detected, justifying molecular testing and opening potential personalized treatment options in current study or compassionate use settings.

## 1. Introduction

Sinonasal melanoma is a rare subtype of melanoma (<1% of all melanomas). While cutaneous melanoma meanwhile has a reasonably characterized mutational profile and various treatment options exist, little is known about sinonasal melanoma. Cutaneous melanoma can be divided into molecular subgroups (*BRAF* mutated, *NRAS* mutated, *NF1* mutated, triple wild-type) and show the typical ultra violet (UV) signature (C > T transition). Several studies have aimed to investigate the mutational profile of sinonasal melanoma. However, most of them are limited to the most commonly known mutated genes in melanoma. Schoenewolf et al. look at *KIT* only, known to be typically altered in mucosal melanoma, and shown to be negatively correlated to immunohistochemical expression [1]; and find one out of 19 tumors to be mutated with strong immunohistochemical staining in up to 25% of the tumor cells. The mutation, however, is synonymous [2]. Other PCR- and Sanger sequencing-based studies find either no or only a low amount of *BRAF*-mutated sinonasal melanoma, few *KIT* mutations, and several *NRAS* mutations [3,4,5,6]. Further investigations include the *TERT* promoter region, which is found to be mutated in 7–8% of sinonasal melanoma, while a known single nucleotide polymorphism (SNP) is found in 82% [7,8]. Only few published studies use a next generation sequencing (NGS) approach, one of them using a 50 gene melanoma panel. Out of 66 patients, 27 patients show one or more mutations, among them, mostly *NRAS* (30%), *BRAF* (7.5%), and *KIT* (4.5%). Only one patient has multiple alterations in *TP53*, *KIT*, *NOTCH1*, *PIK3R1*, and *ERBB2* [9]. Additionally, some of the studies search for gene copy number alterations and find *RREB1* and *MYB* to be lost or *CCND1* to be amplified [4,5]. Another group looks at 29 genes and finds alterations in mainly *NRAS* or *KRAS*, but no *KIT* mutation [10]. The most recent study analyzes a large cohort of 95 sinonasal melanomas [11]. However, only *BRAF*, *NRAS*, *KIT* and *SF3B1* are investigated by Sanger sequencing.

**Figure 1:**
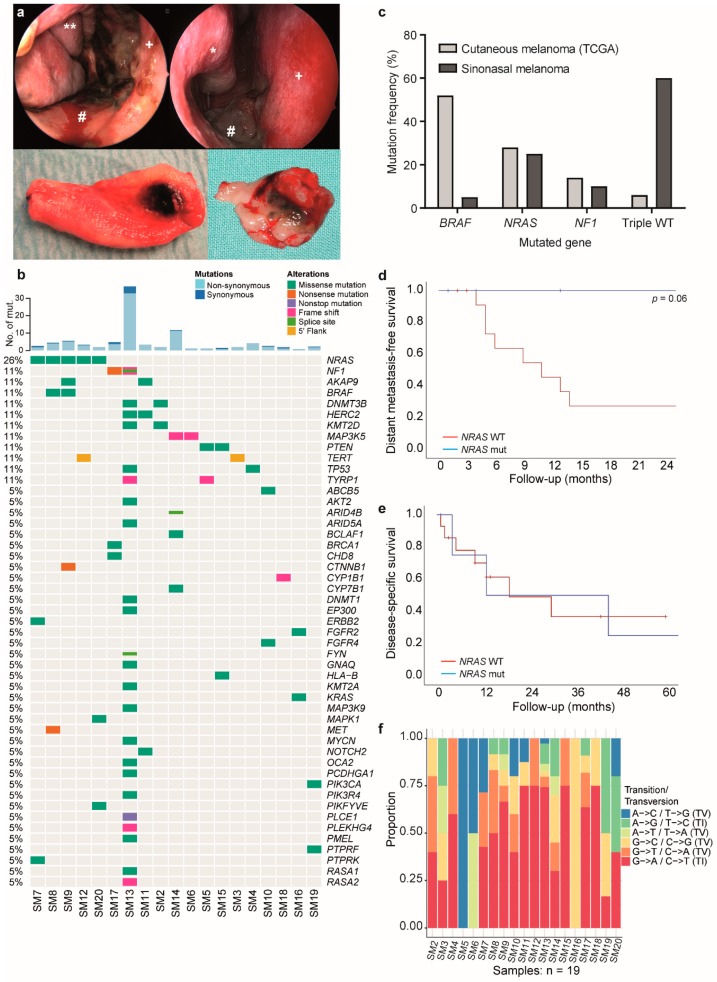
Mutational landscape of sinonasal melanoma. (**a**) Upper panels: Endoscopic photography of a sinonasal melanoma. The dark discoloration of the nasal mucosa with irregular margins is easily recognizable. * Inferior turbinate, ** middle turbinate, # nasal floor, + nasal septum. Lower panels: Surgical specimen after endoscopic removal of sinonasal melanomas. The dark areas of sinonasal melanoma have been excised with sufficient margins of normal mucosa around it. (**b**) Upper part: Analysis of mutational burden with non-synonymous (light blue) and synonymous (dark blue) mutations. Lower part: mutational landscape of the primary sinonasal melanoma cohort. (**c**) Comparison of frequencies of the four molecular subgroups in between cutaneous melanoma (from TCGA) and our primary sinonasal melanoma cohort. (**d**) Survival curve showing distant metastasis-free survival in *NRAS* wild-type (WT) (red) and *NRAS* mutated (blue) patients. (**e**) Survival curve showing disease-specific survival in *NRAS* WT (red) and *NRAS* mutated (blue) patients. (**f**) Transition/Transversion plot of the sinonasal melanoma cohort.

Despite various treatment options for cutaneous melanoma patients, including local and systemic therapies, using targeted or immunotherapeutic agents [12], options for sinonasal melanoma patients are limited. Primary tumors and localized disease are typically treated with surgery and adjuvant radiotherapy [13]. Nowadays, surgery consists of a transnasal endoscopic resection with intraoperative navigation as requested (Figure 1a).

In the metastatic and advanced disease setting, mucosal melanomas are treated according to guidelines for cutaneous melanoma [14]. However, outcome data on systemic treatment are very limited, as mucosal location is often an exclusion criterion in melanoma clinical trials [15]. Furthermore, the disease is comparably rare and immunotherapy studies that did not exclude mucosal melanoma were unable to perform subgroup analyses.

Besides immunotherapy, targeted therapy using specific inhibitors is an option [15]. However, also here, data are limited, partly because targetable mutations in sinonasal melanoma are rare and largely uninvestigated. Some of them showed *KIT* mutations, enabling treatment with imatinib or nilotinib [15]. Others carry *NRAS* mutations, offering the opportunity to use Mitogen-activated protein kinase kinase (MEK) inhibitors [15]. Currently, several ongoing clinical trials evaluate different treatments in mucosal melanoma patients, e.g., ipilimumab/nivolumab combination, or IL2/pembrolizumab combination (NCT03241186, NCT02748564).

In this study, we performed molecular profiling of a cohort of sinonasal primary melanomas using a targeted next generation sequencing (NGS) approach. In addition, we characterized the tumors according to their histological and immunological features. The aim of this study was to further investigate molecular alterations as well as morphological properties and immunogenicity of this rare subtype of melanoma and thereby screen for prognostic markers and possible therapeutic targets.

## 2. Results

### 2.1. Mutation Analysis Reveals a Generally Low Mutational Burden with NRAS Mutation as the Most Frequent Driver

Mutational profiling of 190 genes revealed a low number of mutations in most of the primary sinonasal melanoma samples. MelArray mutational burden reached from 0.7 to 32.9 non-synonymous mutations/Mb (Figure 1b, upper part, light blue) and few synonymous mutations ranging from 0 to 4 mutations/Mb (Figure 1b, upper part, dark blue). Eighteen of 19 patients had a mutational burden ranging from zero to four non-synonymous coding mutations; whereas, one patient had a high mutational burden with 24 non-synonymous coding mutations.

**Figure A1:**
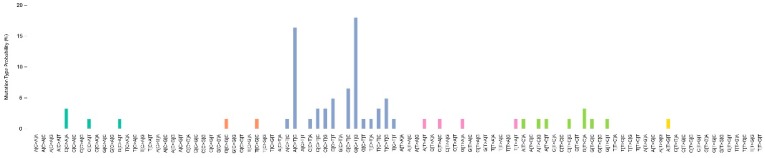
COSMIC signature retrieved from sequencing data of sample SM13.

While *NRAS* was most frequently mutated in our cohort (26%, five patients), other genes were mutated in only one or two patients, illustrating the heterogeneous mutational profile of sinonasal melanoma (Figure 1b, lower part). One of the *NRAS* mutated patients had a (non-canonical) *BRAF* mutation in addition. Two patients had a *NF1* mutation, while all the other 13 patients were triple wild-type according to the molecular classification of melanoma [16]. This result was significantly different to TCGA data of cutaneous melanoma, where *BRAF* mutations are the most frequent driver (Figure 1c). One patient had a *KRAS* mutation, most likely being the oncogenic driver of the disease. The patient with the high mutational burden had a RASopathy profile, showing a *NF1* mutation, together with truncation of *RASA2*, leading to increased activation of Ras [17]. By retrieving the COSMIC (Catalogue Of Somatic Mutations In Cancer) mutational signature from this patient, we found closest similarities with signature 1 and partially signature 15 (Appendix A Figure A1). The detected *GNAQ* mutation (p.Thr54Met) is a variant of unknown significance and is neither reported in the ClinVar database nor found in the TCGA cutaneous or uveal melanoma cohort. Another patient had a subclonal activating *PIK3CA* mutation. No patient had mutations in *KIT*. Two patients showed *TERT* promoter mutations, with one being a so far unknown mutation.

We analyzed the clinical data of patients with and without driver mutations and did not find any significant correlation with local recurrence rates or survival (Log-rank test, *p* = 0.125 and *p* = 0.776). However, patients with *NRAS* mutations showed a trend towards prolonged distant metastasis-free survival (Figure 1d) (Log-rank test, *p* = 0.06), but no difference in disease specific survival (Figure 1e, *p* = 0.9), while the two groups (*NRAS* mutant vs. *NRAS* wild-type) showed equal distribution of age, sex, and TNM stage.

Furthermore, we analyzed our data for UV signature mutations (mostly C > T transition) and found a generally lower number of C > T transitions compared to published data for cutaneous melanoma. All samples had C > T transitions below 75%, which was the median percentage found in the TCGA for cutaneous melanoma. Seven samples had more than 60% C > T (Figure 1f).

### 2.2. Presence or Absence of DNA Repair Gene Alterations Divide the Cohort into Different Groups

**Figure 2:**
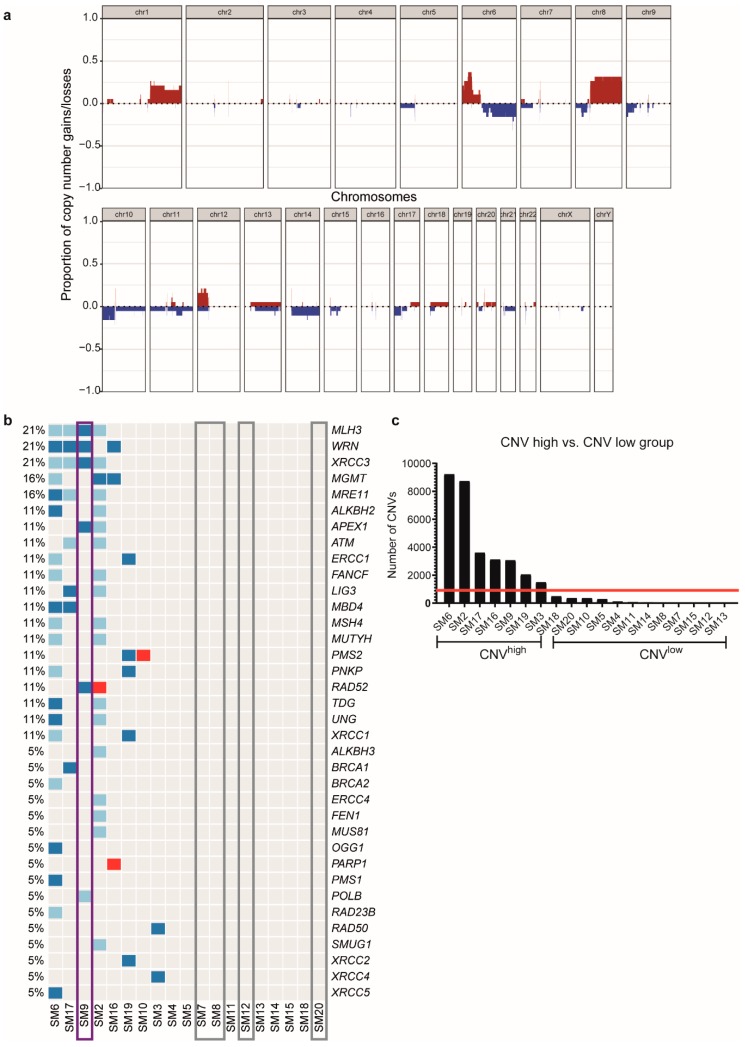

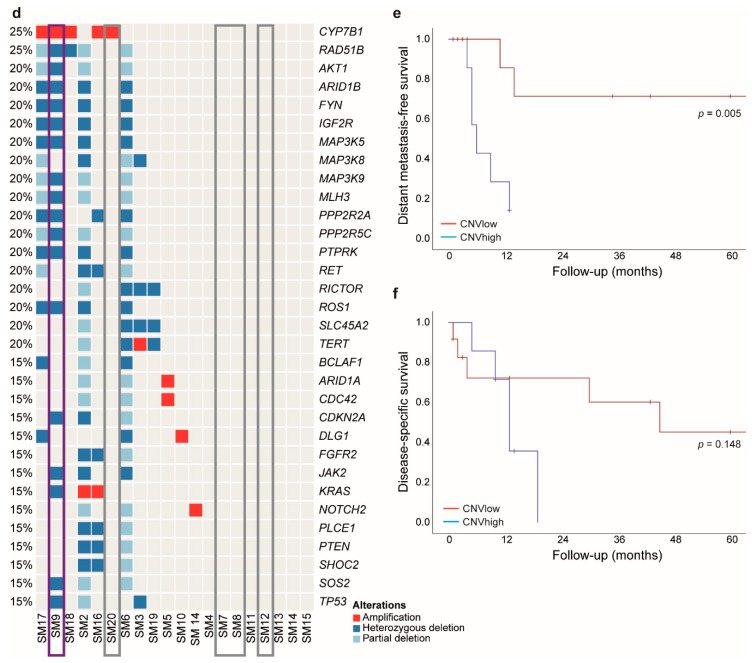
Genome-wide copy number assessment of the primary sinonasal melanoma cohort. (**a**) Global copy number gains (red) and losses (blue). (**b**) Copy number alterations in DNA repair genes. Grey boxes: *NRAS* mutant patients, purple box: *BRAF*/*NRAS* double mutated patient. (**c**) Amount of copy number variations (CNVs) across the cohort. Red bar: 1000 CNVs. (**d**) Copy number alterations in the most frequently altered MelArray genes. Grey boxes: *NRAS* mutant patients, purple box: *BRAF*/*NRAS* double mutated patient. Red: amplifications, light blue: partial deletions, dark blue: heterozygous deletions. (**e**) Survival curve showing distant metastasis-free survival in CNV^high^ (blue) and CNV^low^ (red) patients. (**f**) Survival curve showing disease-specific survival in CNV^high^ (blue) and CNV^low^ (red) patients.

Genome-wide assessment of copy number alterations in our cohort of sinonasal melanoma revealed amplifications of parts of chromosomes 1, 6, 8, and 12 and partial losses of chromosomes 6, 8, 10, 14, and 17 (Figure 2a). Furthermore, we detected two different groups (Figure 2b), one with alterations in DNA repair genes, and one without. By looking into further differences between those two groups, it was evident that the group with copy number variations (CNVs) in DNA repair genes had a generally high amount of CNVs (>1400; hereafter named CNV^high^), while the other group had only few CNVs (<850; hereafter named CNV^low^) (Figure 2c). All of the CNV^high^ patients had partial or heterozygous loss of one or more genes involved in DNA repair, such as *ATM*, *MLH3*, *BRCA1/2*, *PMS2* or amplification of *PARP*. In contrast, all but one patient of the CNV^low^ group had no changes in copy numbers of DNA repair genes, except for a patient with a *PMS2* amplification (Figure 2b).

### 2.3. Low CNV Numbers Are Associated with NRAS Mutations and Prolonged Distant Metastasis-Free Survival

Interestingly, all patients with *NRAS* mutations (Figure 2b, grey) had low levels of CNVs and no alterations in DNA repair genes, except for the patient with the *BRAF*/*NRAS* double mutation (Figure 2b, purple), who had a high number of CNVs and various alterations in DNA repair genes. Furthermore, while all *NRAS* mutant patients (Figure 2d, grey) had either no alteration or only an amplification in the 190 MelArray genes, the *BRAF*/*NRAS* mutated patient (Figure 2d, purple) showed numerous deletions as well. Correlation with clinical data revealed a prolonged distant metastasis-free survival of patients in the CNV^low^ group (Figure 2e; *p* = 0.005). There was no difference in overall survival (Figure 2f; *p* = 0.148).

### 2.4. Fusions

Investigation of 28 canonical fusion breakpoints in *ALK*, *BRAF*, *MAP3K8*, *MET*, *NTRK1*, *PRKAR1A*, *RAF1*, *RET*, and *ROS1* did not reveal any fusion events in our cohort.

### 2.5. Oncogenic Alterations Are Mostly Associated with MAPK or PI3K/mTOR Pathway

Overall, we could identify molecular alterations that may contribute to tumorigenesis in most of the samples. These alterations were mainly associated with activation of MAPK and PI3K/mTOR signaling pathways. Furthermore, alterations affecting cell cycle and DNA repair were evident (Table 1).

### 2.6. Histological Evaluation Shows an Association of Monomorphic Features with Worse Outcome

**Figure 3:**
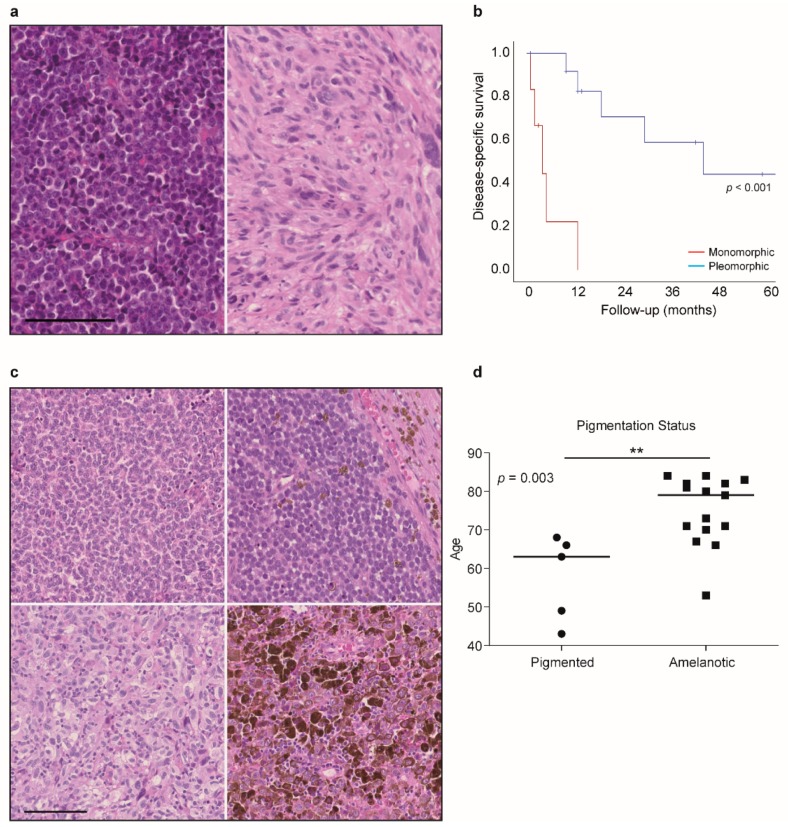
Histological characteristics of the primary sinonasal melanoma cohort. (**a**) hematoxylin and eosin (H and E) staining of a representative case of primary sinonasal melanoma with monomorphic (left) and pleomorphic (right) histology. (**b**) Survival curve showing disease-specific survival in patients with monomorphic (red) and pleomorphic (blue) histology. (**c**) H and E staining of a representative case of monomorphic amelanotic tumor (upper left), monomorphic pigmented tumor (upper right), pleomorphic amelanotic tumor (lower left), and pleomorphic pigmented tumor (lower right). (**d**) Graph showing an association of tumor pigmentation and age. Data are displayed with median. ** *p* < 0.01. Scale bar in all panels 100 μm.

Besides molecular profiling, all tumors were analyzed according to morphology (monomorphic vs. pleomorphic), presence of apoptosis, and presence of pigmentation. There was no correlation of these features with either mutation or CN status. However, prolonged disease-specific survival was clearly associated with a pleomorphic morphology (Log-rank test, *p* < 0.001) (Figure 3a,b). Furthermore, all patients who received palliative care at first presentation turned out to have melanomas with monomorphic appearance. The only patient who received curative treatment died within six months after diagnosis; whereas, all other patients in the curative arm had a pleomorphic morphology and had a longer disease-specific survival. There was no significant difference in sex or age of the patients in the two groups (Log-rank test, *p* > 0.05).

Only one of the monomorphic melanoma tumors was pigmented. Presence or absence of apoptosis or pigmentation had no impact on clinical properties. However, amelanotic melanoma were more common in older patients (Mann Whitney U, *p* = 0.003). Amelanotic tumors were confirmed to be melanoma by immunohistochemical positivity of S100, HMB-45, and MelanA (Table 2). The median age of patients with pigmented melanoma was 63.0 years (interquartile range (IQR) 46–67); whereas, the mean age of patients with amelanotic melanoma was 79.5 years (IQR 69.25–89.25) (Figure 3c,d).

### 2.7. Immunohistochemistry Reveals Mostly PD-L1 Negativity and Infiltration of T-Cells

All primary tumors were stained for PD-L1, CD4 and CD8 to assess PD-L1 status and T-cell infiltration. Most of the tumors were negative for PD-L1, whereas most patients had some positivity for PD-L1 in immune cells. Only one tumor exhibited strong staining, while three others showed a weak to intermediate staining. The patient with the high mutational burden showed no PD-L1 expression in the tumor, but intermediate expression in the immune cells. Most of the tumors had intermediate to strong infiltration of CD4+ and CD8+ cells. Three tumors did not show any T-cell infiltration or PD-L1 staining of immune cells and tumor. These factors did not show any correlation with oncological outcome measures.

### 2.8. Immunotherapy is an Option for Sinonasal Melanoma Patients

**Figure 4:**
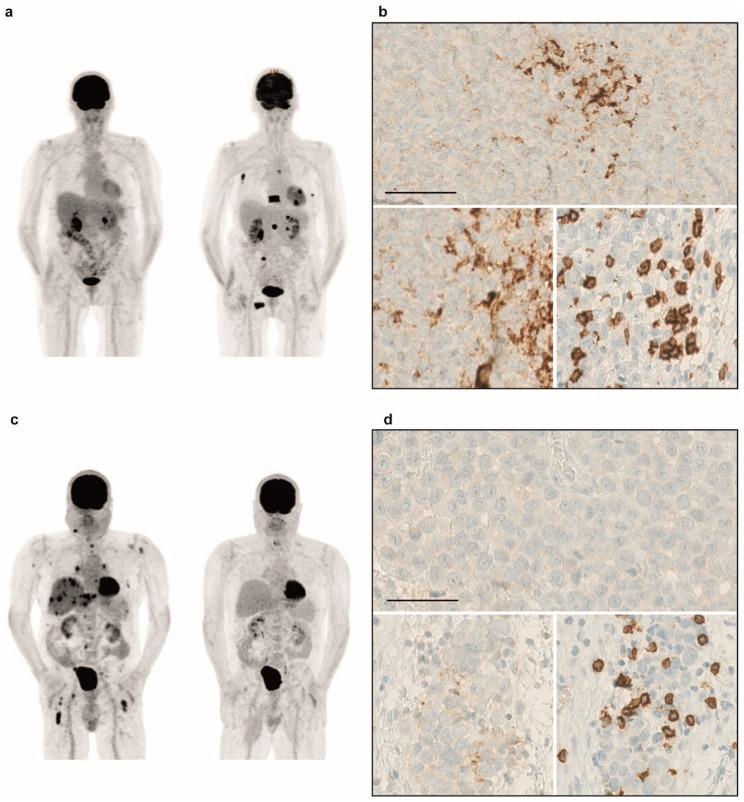
FDG-PET/CT and immunohistochemical characteristics of non-responder and responder with sinonasal melanoma to immunotherapy. (**a**) FDG-PET/CT scan of a sinonasal melanoma patient, progressing under immunotherapy (left: before treatment; right: progression). (**b**) Immunohistochemical expression of PD-L1 in the tumor (upper panel), and in the immune cells (lower left), the latter corresponding to CD4 positive cells in the same region (lower right). (**c**) FDG-PET/CT scan of a sinonasal melanoma patient, responding to immunotherapy (left: before treatment; right: complete response). (**d**) No recognizable immunohistochemical expression of PD-L1 in the tumor cells (upper panel), and virtually absent PD-L1 expression in the immune cells (lower left), the latter corresponding to CD4 positive cells in the same region (lower right). Scale bar in all histology panels 50 μm.

Current treatment options are rare as there are no targeted therapies for sinonasal melanoma. While patients with systemic disease were treated with chemotherapy or sorafenib in the past, today, immunotherapy is an option. Three patients of our cohort received immunotherapy, with either nivolumab or pembrolizumab monotherapy or a combination of ipilimumab and nivolumab. While one patient receiving ipilimumab/nivolumab combo responded, treatment had to be stopped due to side effects. The other patient treated with the combination therapy received four cycles of the combination and proceeded with 21 cycles of nivolumab monotherapy. Both patients responded to treatment. The patient treated with pembrolizumab did not respond. Interestingly, the two responding patients in our cohort were negative for PD-L1 in the tumor (TC0), while the non-responder showed some expression (i.e., TC1). Furthermore, responding patients had a low PD-L1 score in the immune cells (IC0 or IC1), whereas the non-responder had an IC3 (Figure 4). All three patients had low levels of CD4 infiltration and low or high CD8 infiltration. There was no difference in TMB between responders and the non-responder. While the non-responder had high CNV numbers (2049 CNVs), the responders had either high (3120 CNVs) or low CNV (849 CNVs) numbers. However, the small sample size and different treatment regimens do not allow for proper statistical analysis.

### 2.9. Molecular Alterations Provide Opportunities to Experimental Therapy

After progression under approved therapies, there may be options to include patients into clinical studies or compassionate use programs due to their molecular alterations. In our cohort, we detected several alterations that may allow treatment apart from approved therapies. Patients with alterations in *NF1* could be included into clinical trials investigating the effect of the MEK inhibitor cobimetinib (NCT02639546), the mTOR inhibitor temsirolimus (e.g., NCT03297606), a SHP-2 inhibitor (NCT03634982) or the pan-RAF inhibitor LXH254 (NCT02607813). Alterations in *PIK3CA* or *PTEN* allow inclusion into clinical trials with mTOR inhibitors everolimus or temsirolimus (NCT02029001, NCT03297606). Furthermore, patients with *PIK3CA* alteration can be included into studies investigating PI3K inhibitors (e.g., NCT03006172) and patients with *PTEN* alterations could be treated with the AKT inhibitor ipataserib in combination with atezolizumab in clinical trials (NCT03673787). Other trials are investigating the effect of the CDK4/6 inhibitor palbociclib in patients with *CDKN2A* alterations (e.g., NCT02693535, NCT03297606). Loss of *BRCA1* is an inclusion criterion for clinical trials evaluating the effect of a PARP inhibitor in combination with immunotherapy (e.g., NCT03330405, NCT02660034).

## 3. Discussion

Our finding of low mutational burden in sinonasal melanoma is consistent with published data about mucosal melanoma that had a mean mutational burden of 2.64 mutations/Mb ranging from 0.54–15.25 mutations/Mb [18] and showed a significant difference to cutaneous melanoma that usually have a very high mutational burden [19,20].

While cutaneous melanoma can be divided into four molecular subgroups (*BRAF*-, *NRAS*-, *NF1*- mutated or triple WT) [19], our cohort of sinonasal melanoma did not show such distribution. However, the frequency of *NRAS* mutations seemed to be similar in cutaneous and sinonasal melanoma (26% this study vs. 28% TCGA vs. 26% Hodis et al.) [19,20]. Other mutations known from cutaneous melanoma were rare, which is in line with previous reports [6,9]. As also reported previously, the *BRAF* mutation was not a p.V600 hotspot mutation [4,5]. Mutations in *KIT*, which are commonly found in acral melanoma and mucosal melanoma of other localizations [18,21], were not present in our cohort of sinonasal melanoma. The presence of *KIT* mutations is controversially reported, as there are both observations of low frequency and an absence of *KIT* mutations in sinonasal melanoma [4,5,6,7,9,22]. Furthermore, only one subclonal variant of unknown significance in *GNAQ* and no *GNA11* mutations, usually present in uveal melanoma, were found [23]. Interestingly, one patient showed a RASopathy profile with *NF1* truncation and mutations in RASopathy genes like *RASA1* and *RASA2*, which is known from cutaneous melanoma [17]. To the best of our knowledge, this profile has not yet been described in sinonasal melanoma. *NF1* mutated melanoma was previously associated with a higher mutational burden and elevated C>T transition [24]. Canonical *TERT* promoter mutations were previously found in 8% of sinonasal melanoma, with 82% of tumors having –245G>A SNPs [8]. Our cohort had one patient with a canonical –124C>T mutation and one patient with a –185C>G mutation, which was previously unknown. *SF3B1* mutations, reported at low frequencies in other cohorts [11], were not present in our cohort. A study from Cosgarea et al. investigate mucosal melanomas using a NGS panel including 15 head and neck primary tumors. However, the exact location of those head and neck mucosal melanomas are not further specified, so probably sites other than sinonasal tract have been included (e.g., oral cavity). Nevertheless, they find similar results with *NRAS* mutations as the most frequent driver and no evidence of *KIT* mutations [10].

For cutaneous melanoma, it was previously shown that *NRAS* mutations are associated with shorter distant metastasis-free survival compared to *BRAF* mutated patients, but there is no difference between *NRAS* mutated and *BRAF*/*NRAS* WT patients [25]. Likewise, we did not find differences in overall survival between *NRAS* mutant and wild-type patients, but a trend towards longer distant metastasis-free survival in *NRAS* mutated patients. Amit et al. reported no association of *NRAS* mutation and disease-free survival or distant-metastasis free survival (*p* = 0.31 and 0.57) [9]. Furthermore, in cutaneous melanoma, a correlation of *NRAS* mutation and higher age was found [26].

By analyzing UV signature mutations, we found both samples with low C>T transition and samples with higher C>T transition. However, all samples were below the median percentage of 75% C>T transition found for cutaneous melanoma in the TCGA [19]. Actually, 76% of primary tumors and 84% of metastatic samples had UV signatures in the TCGA; whereas, only 36% of our sinonasal melanomas had a C>T transition above 60%. Previously published data about mucosal melanoma (including sinonasal) showed 60% UV signature in mucosal melanoma compared to 70% in cutaneous melanoma [22].

Investigation of copy number changes revealed a high amount of CNVs in a number of samples, which fits to a finding by Curtin et al., who report a higher degree of copy number alterations in acral and mucosal melanoma compared to cutaneous ones [27]. By comparing chromosomal aberrations, we found similar alterations, e.g., gains of chromosome 1q, 6p, 17q or losses of chromosome 8p, 10, and 11p. Likewise, a study on 14 sinonasal melanoma that were investigated by CGH array, finds gains of chromosome 1q, 6p, and 8q, similar to our results [28]. However, approximately half of our samples had a low number of CNVs and survival analysis showed a prolonged distant metastasis-free survival in CNV^low^ patients, which was, to our knowledge, not yet reported elsewhere.

Loss of *MYB* was found in four of our cases. Previously, such a loss was observed by *MYB* FISH [4]. Furthermore, they show a loss of PTEN and CDKN2A (p16) protein by using immunohistochemistry. Accordingly, four of our samples had a loss of *PTEN* and/or *CDKN2A*, respectively. While Chraybi et al. found *CCND1* amplifications in several cases [5], our results showed only one case with a loss and one case with an amplification.

Fusion events in melanoma are rare and sometimes result in drug resistance [29]. Usually they occur in tumors without driver mutations [30]. We investigated 28 canonical fusion breakpoints in seven genes known from rearrangements in melanoma. However, we did not detect any gene fusions.

While specific mutations do not seem to affect the outcome of the disease, these alterations could serve as potential targets for systemic therapy in metastatic sinonasal melanoma. The BRAF/MEK inhibitor combination is already approved for melanoma with *BRAF* mutation. Other inhibitors could be made available via clinical studies or compassionate use programs. Patients with defects in the DNA repair machinery may respond to PARP inhibitor treatment, as previously shown for prostate cancer patients [31]. *NF1* mutated melanomas may respond to MEK inhibitor treatment as suggested by preclinical studies and a case report [32]. A clinical trial will investigate the effect of everolimus, sorafenib or trametinib in *NF1*-mutated melanoma (NCT02645149). In the NCI-MATCH trial, patients with *NF1* mutations are treated with trametinib. However, no results have been published yet, as completion of the trials is expected for mid-2022. Furthermore, clinical trials are investigating the impact of CDK4/6 inhibitors in patients with *CDKN2A* loss and the impact of mTOR inhibitors on patients with *PIK3CA* or *PTEN* alterations (NCT02645149, NCT03297606). However, due to the very low incidence of sinonasal melanoma, there are no specific clinical studies or case reports on such experimental therapies. Mutational profiling in sinonasal melanoma patients will identify the above-mentioned targets and may allow inclusion of patients into trials for solid tumors.

To our knowledge, an association of pleomorphic morphology and prolonged disease specific survival compared to tumors with monomorphic histology, as found here, is not reported elsewhere. Similar to Amit et al., we did not find any association of morphology and mutation status [9]. We saw amelanotic tumors being associated with higher age of the patients, but we did not observe a correlation with pigmentation and survival, even though only one of the monomorphic melanomas was pigmented. These results contradict a study, that shows an amelanotic subtype of sinonasal melanoma is associated with worse outcome [33], however, the study cohort is all Chinese patients, and therefore a different ethnic background from our samples. For cutaneous melanoma, a large cohort is analyzed and a poor survival of amelanotic melanoma is shown compared to pigmented tumors [34].

PD-L1 staining of the primary sinonasal melanomas revealed a negative result in most patients, which is in line with published data about mucosal melanoma that shows significantly less PD-L1 expression compared to cutaneous melanoma [22]. However, the relevance of PD-L1 expression in melanoma is not that clear, as the combination of ipilimumab and nivolumab was shown to be effective in both PD-L1 positive and negative patients [35].

Three patients showed a loss of *CD274*, the PD-L1 gene, with no expression of PD-L1 in the immunohistochemical staining. Although this might lead to failure of therapeutic response, the number of cases was too small to make further conclusions. A report in oral squamous cell carcinoma showed that *CD274*-amplified tumors has a significantly higher frequency of positive PD-L1 staining than cases without amplification [36].

## 4. Materials and Methods

### 4.1. Patient Samples

We included 19 patients who were diagnosed and treated at the University Hospital Zurich from 2006–2018 and that had resected, primary tumors available. The local ethics review board approved the study (BASEC 2016.00162, including amendment from 03.05.2018). All patients treated in 2016 and afterwards signed written informed consent approved by the local ethics review board (EK647/800) in addition to the general institutional consent.

### 4.2. Collection of Clinical Data

A retrospective chart review of all patients with sinonasal melanoma treated at the Department for Otorhinolaryngology—Head and Neck Surgery of the University Hospital Zurich, was performed (Table 3). We examined charts to obtain detailed demographic and clinical data (gender, age, TNM stage, lymph node involvement, orbit and skull base invasion, treatment modalities, follow-up, and recurrence). We included only cases with histologically and immunohistochemically verified sinonasal melanoma in the analysis. A Swiss Medical Association board-certified head and neck pathologist (N.J.R) reviewed all cases to ensure the accuracy of the diagnosis. Staging was performed according to the Union International Contre le Cancer (UICC), TNM Staging for sinonasal cancer, 8th edition 2018 [37]. All patients were presented at the local multidisciplinary tumor board and treatment recommendation was based on the available guidelines and literature at the time of the patients’ accrual.

### 4.3. Sample Preparation

Patient material was obtained from formalin-fixed paraffin-embedded (FFPE) tissue. One 2 µm section was stained with hematoxylin and eosin (H and E) and a pathologist (N.J.R.) marked the tumor area as well as regions of normal tissue. Punch biopsies of 0.4 mm in diameter were taken from both areas.

### 4.4. DNA Isolation

DNA of normal and tumor tissue was isolated using the Maxwell 16 FFPE Tissue LEV DNA Purification Kit (Promega, Madison, WI, USA) and quantified using a fluorometric assay (Qubit, Thermo Fisher Scientific, Waltham, MA, USA).

### 4.5. Library Preparation

The KAPA HyperPlus Kit was used to fragment the DNA and build sequencing libraries. Unique sequencing adapters were ligated to the libraries to allow pooling of up to 12 libraries for target capture, which was performed using a customized probe set [38] (manuscript submitted) by Roche NimbleGen (Basel, Switzerland). 

### 4.6. Sequencing

Of the samples, 16–18 were sequenced paired-end (150 bp) on one lane of a HiSeq4000 Illumina machine (Illumina, San Diego, CA, USA).

### 4.7. Analysis

A customized pipeline and open source software were used to analyze the data. After demultiplexing, fastq files went through quality control using Picard. Read trimming was performed using skewer [39] and bwa (Burrows-Wheeler Aligner) alignment [40]. GATK4 and MuTect2 [41] were used for generation of vcf files. CNV analysis was done using Sequenza to derive logR values [42].

### 4.8. (Immuno-)Histological Staining and Evaluation

Formalin-fixed and paraffin-embedded (FFPE) specimens were examined on 2 µm hematoxylin and eosin (H and E) stained sections. The Ventana Benchmark automated staining system was used for immunohistochemical staining on 2 µm sections. For PD-L1 staining the anti-PD-L1 (E1L3N) antibody (1:100, Cell Signaling Technology, Cambridge, United Kingdom) was used. Detection was performed with optiView DAB-kit (Ventana). For CD4 staining a polyclonal anti-CD4 antibody (1:100, R&D Systems, McKinley, MN USA) was used. CD8 staining was performed using a monoclonal anti-CD8 (4B11) antibody (Bio-Rad Laboratories; 1:100). Detection was performed with rabbit-anti-goat horseradish peroxidase. Histological features (morphology, pigmentation, apoptosis) and immunohistochemical staining for CD4, CD8, and PD-L1 were evaluated by an experienced head and neck pathologist (N.J.R.). PD-L1 staining was scored according to the scheme established for lung cancer [43]: tumor cells: TC0 = negative, TC1 = 1–5%, TC2 = 5–50%, TC3 ≥ 50%; immune cells: IC0 = negative, IC1 = 1–5%, IC2 = 5–10%, IC3 ≥ 10%. Staining of melanocytic markers was performed as described previously [44].

### 4.9. Statistics

For continuous variables, median, interquartile range (IQR), or standard deviation (SD) were given. To compare the distribution among samples, the non-parametric Mann Whitney U test was used for two samples. Binary variables were associated in contingency tables using the two-tailed Fisher exact test. Survival curves were plotted according to Kaplan–Meier and the Log-rank test was used to compare factors. A *p*-value lower than 0.05 was considered to indicate statistical significance. Statistical analyses were performed using SPSS^®^ 25.0.0.0 software (IBM^®^, Armonk, NY, USA).

## 5. Conclusions

This study shows the importance of molecular profiling in sinonasal melanoma as it revealed possible clinical trial opportunities and compassionate use programs for these patients with limited treatment options. Furthermore, it has prognostic value, as, high number of CNV load is associated with reduced distant metastasis-free survival, whereas *NRAS* mutations are associated with a low CNV load. Notably, *KIT* mutations were absent from our cohort. Additionally, a monomorphic histology was associated with worse outcome of the disease (Table 4), which supports the notion that a combination of next generation sequencing data and immunohistochemistry are important tools for clinical decision support for sinonasal melanomas.

## Figures and Tables

**Table 1:** Main molecular alterations and affected pathways.

Sample	Alteration	Pathway/Function
SM2	*KRAS* amplification, *NF1*/*PTEN*/*CDKN2A* loss	MAPK pathway, PI3K/mTOR pathway, cell cycle
SM3	*TP53* loss	Tumor suppressor
SM4	*TP53* p.Arg273Cys (subclonal)	Tumor suppressor
SM5	*PTEN* p.Leu108Arg	PI3K/mTOR pathway
SM6	*MAP3K5* p.Gly39AlafsTer142 (subclonal), *MAP3K5*/*PTEN*/*CDKN2A* loss	p38 pathway, PI3K/mTOR pathway, cell cycle
SM7	*NRAS* p.Gln61His	MAPK pathway
SM8	*NRAS* p.Gly61Lys	MAPK pathway
SM9	*BRAF* p.Asp594Asn, *NRAS* p.Gly12Asp, *TP53*/*CDKN2A* loss	MAPK pathway, pathway cell cycle
SM10	-	-
SM11	-	-
SM12	*NRAS* p.Gln61Lys, *TERT* prom. -124C>T	MAPK pathway
SM13	*NF1* p.Leu307Ter	MAPK pathway
SM14	*MAP3K5* p.Pro47ValfsTer56, *MAP3K5* p.Gly39AlafsTer142 (both subclonal)	p38 pathway
SM15	*PTEN* p.Arg74Ile (subclonal)	PI3K/mTOR pathway
SM16	*KRAS* p.Gly12Ala, *KRAS* amplification, *PTEN* loss	MAPK pathway, PI3K/mTOR pathway
SM17	*NF1* p.Gln1520Ter, *NF1* loss, *BRCA1* loss, *BRCA1* predictive damaging VUS	MAPK pathway, DNA repair
SM18	-	-
SM19	*PIK3CA* p.Glu545Gly	PI3K/mTOR pathway
SM20	*NRAS* p.Gln61Arg	MAPK pathway

**Table 2:** Immunohistochemical staining of melanocytic markers. The staining was rated as “+” (positive), “(+)” (partially positive), “−” (negative) or “N/D” (not determined).

Sample	Pigmentation Status	S100	HMB-45	MelanA	SOX10
SM2	pigmented	+	N/D	+	N/D
SM3	amelanotic	+	N/D	+	N/D
SM4	amelanotic	(+)	+	+	N/D
SM5	amelanotic	+	N/D	+	N/D
SM6	pigmented	+	N/D	+	N/D
SM7	amelanotic	+	(+)	+	N/D
SM8	amelanotic	+	(+)	+	N/D
SM9	amelanotic	+	(+)	+	N/D
SM10	amelanotic	+	+	+	N/D
SM11	amelanotic	−	+	(+)	+
SM12	pigmented	+	N/D	+	N/D
SM13	pigmented	+	+	+	N/D
SM14	amelanotic	+	+	+	N/D
SM15	amelanotic	+	+	+	N/D
SM16	amelanotic	(+)	+	(+)	+
SM17	amelanotic	+	+	+	N/D
SM18	amelanotic	+	+	+	N/D
SM19	pigmented	+	+	+	N/D
SM20	pigmented	+	+	+	N/D

**Table 3:** Description of the sinonasal melanoma patient cohort.

Characteristics	Distribution in the cohort
Gender	n (%)
Male	8 (42%)
Female	11 (58%)
Age at diagnosis	Median (range)
Male	72 (53–84)
Female	71 (49–84)
Total	71 (43–84)
Clinical classification of the tumor	n (%)
T3	11 (58%)
T4	8 (42%)
Pigmentation status of primary tumor	n (%)
Pigmented	6 (32%)
Amelanotic	13 (68%)
Morphology of primary tumor	n (%)
Monomorphic	6 (32%)
Pleomorphic	13 (68%)

**Table 4:** Overview of major findings.

	Distant Metastasis-Free Survival		Disease-Specific Survival	
	**Median (SE)**	***p*-Value**	**Median (SE)**	***p*-Value**
***NRAS* Status**				
WT	13.1	0.049	19.0 (10.7)	0.921
Mutated	60.0		13.0 (20.5)	
**CNV Status**				
CNV^high^	-(-)	0.005	13.0 (1.6)	0.148
CNV^low^	6.0 (1.3)		45.0 (17.95)	
**Morphology**				
Monomorphic	5.0 (-)	0.941	4.0 (2.0)	0.000082
Pleomorphic	13.0 (2.6)		45.0 (17.95)
